# Near-Infrared Fluorescent Probes for the Detection of Cancer-Associated Proteases

**DOI:** 10.1021/acschembio.1c00223

**Published:** 2021-07-27

**Authors:** Jamie I. Scott, Qinyi Deng, Marc Vendrell

**Affiliations:** † Centre for Inflammation Research, The University of Edinburgh, EH16 4TJ Edinburgh, United Kingdom

## Abstract

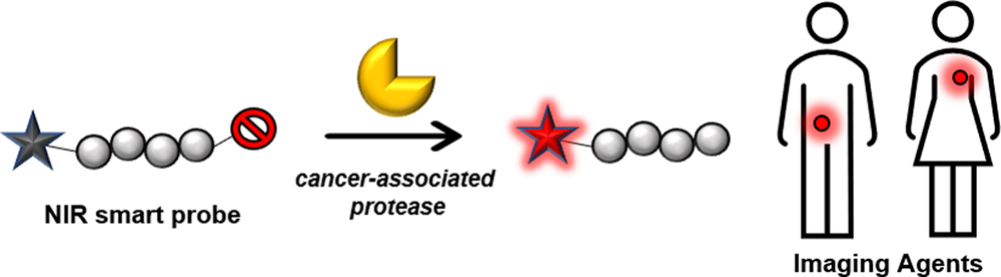

Proteases are enzymes capable of catalyzing protein breakdown, which is critical across many biological processes. There are several families of proteases, each of which perform key functions through the degradation of specific proteins. As our understanding of cancer improves, it has been demonstrated that several proteases can be overactivated during the progression of cancer and contribute to malignancy. Optical imaging systems that employ near-infrared (NIR) fluorescent probes to detect protease activity offer clinical promise, both for early detection of cancer as well as for the assessment of personalized therapy. In this Review, we review the design of NIR probes and their successful application for the detection of different cancer-associated proteases.

## Introduction

The ability to acquire images in vivo in intact organisms provides a wealth of physiological and pathological information that is not available when analyzing cells or tissues *ex vivo*. This can allow clinicians to detect tumors earlier, monitor the formation of metastases, and evaluate the efficacy of anticancer treatments. On a more mechanistic level, biomedical imaging can serve as a tool for translating clinically relevant animal models of cancer into human studies.^1−4^ Currently, clinical imaging is mostly derived from computed tomography (CT), magnetic resonance imaging (MRI),^5,6^ positron emission tomography (PET),^7^ and single-photon emission computed tomography (SPECT).^8^ While providing useful clinical data, these imaging modalities are hampered by the need for ionizing radiation (PET and SPECT), poor spatial resolution, and limited sensitivity. Fluorescence imaging circumvents these issues by using nonionizing light while providing enhanced spatiotemporal resolution.^9−11^ Furthermore, the versatility of fluorescent chemical labels offers multiple options to achieve optimal contrast and obtain functional readouts of specific biomolecules.^12−16^

**Figure 1:**
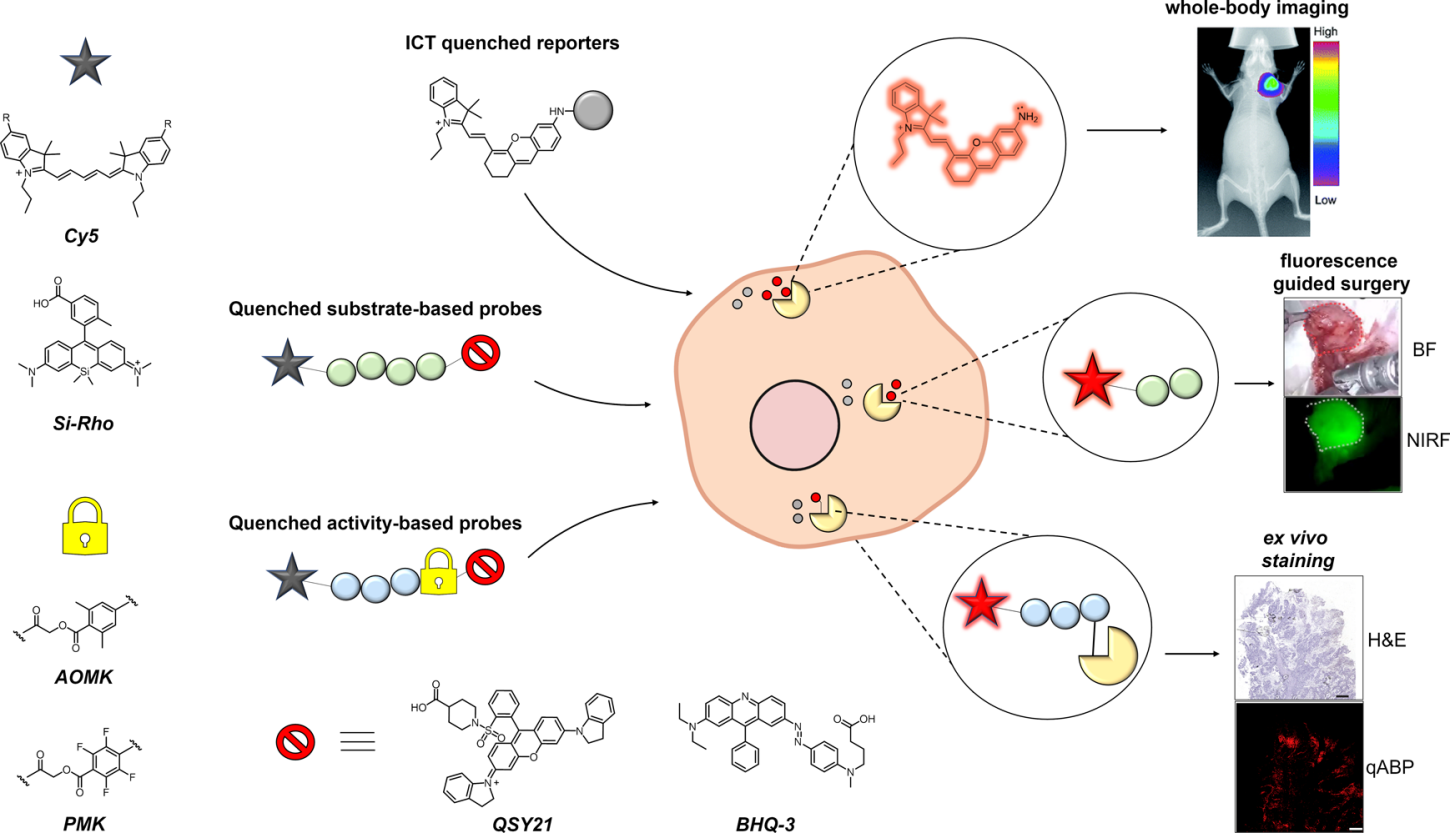
Generic representation of different NIR activatable probes, their building blocks, activation mechanisms, and biological applications. (Top) Fluorescence quenched by an internal charge transfer (ICT) effect that is released upon proteolytic cleavage of the amino acid residue. A specific application of the ICT quenched probe HCAL is shown in whole body imaging of leucine aminopeptidase (LAP) activity in mice bearing HepG2 xenografted tumors. Adapted with permission from ref (37). Copyright 2017 Royal Society of Chemistry. (Center) Fluorescence quenched by a FRET mechanism between the fluorophore and the quencher and restored following enzymatic substrate cleavage. Application of the agent 6QCNIR for detection and fluorescence-guided surgical removal of 4T1 mouse breast tumors using a da Vinci surgical instrument. The image shows white light illumination of tumor and tumor bed (top), as well as the corresponding fluorescence image detected using the Firefly camera system (bottom). Adapted with permission from ref (117). Copyright 2015 American Chemical Society. (Bottom) Quenched activity-based probes covalently modify their target enzyme, with substrate hydrolysis removing the quencher and forming a fluorescent probe–protease conjugate. A representative application highlights *ex vivo* biopsy tissue staining of cathepsins in human polyps after administration of BMV109 with the top image being H&E stain and the bottom image being NIR fluorescence. Adapted with permission from ref (109). Copyright 2015 Elsevier. Star represents NIR fluorophores. The yellow lock represents electrophilic trap groups, and the red stop sign represents commonly used quencher molecules.

NIR fluorescence imaging can achieve superior penetration depth through tissues when compared to fluorescence emission in other regions of the visible spectrum. NIR light can penetrate 5–8 mm beneath human skin vs 1–2 mm for green light.^17−21^ NIR light falls within an optimal biological window where the proteins and biomolecules found in tissues display reduced light scattering coefficients, decreased absorbance, and minimal autofluorescence. These features result in increased signal-to-noise ratios with deeper tissue penetration. Broadly categorizing, there are two classes of NIR probes depending on the factors that trigger their fluorescence emission. The first are always-on fluorophores, which can be used for cell tracking or to feature vascular distribution in heavily vascularized tumors.^22^ Indocyanine Green (ICG) is the main FDA-approved NIR fluorophore to date and has been utilized in clinical studies to improve tumor resection by enhancing the contrast between tumor and healthy tissue. While the staining with ICG can provide significant benefit to surgeons, it cannot provide activity readouts of cellular processes within the tumor microenvironment. In light of this, the activity of proteases has emerged as a biomarker of cancer progression with many tumors showing elevated levels of proteolytic enzymes in early stages of the disease. Proteases are interesting targets for activatable NIR probes, an alternative class of fluorophores which change their spectral properties upon target engagement, because they can be designed to react with specific enzymes.^23−25^ Activatable probes featuring specific peptide sequences can be tailored to individual proteases so that enzymatic activities within tumors can be monitored by their fluorescence readouts (Figure 1). In this Review, we will review the progress in the past 10 years in the design of NIR probes for imaging different families of proteases associated with the progression of cancer, including aminopeptidases, cysteine proteases, serine proteases, and matrix metalloproteinases (MMPs). The chemical structure, protease target, and excitation/emission wavelengths for all probes discussed are listed in the Supporting Information.

### Aminopeptidases

Aminopeptidases are a group of enzymes that cleave specific amino acids at the N-terminus of peptides or proteins. There are reports that link the expression of aminopeptidases to cancer,^26,27^ presenting themselves as potential imaging biomarkers. Targeting the hydrolytic properties of aminopeptidases offers an appealing strategy for molecular imaging via the use of fluorogenic probes. In most cases, the design of such NIR activatable probes involves the conjugation of the amino acid recognized by the enzyme to a free amino group of a NIR fluorophore. When the probe is intact, intramolecular charge transfer (ICT) renders the probe in a nonemissive state, whereas the enzymatic cleavage of the amino acid results in a spectral shift and large emission increase from the fluorophore. This strategy is useful for targeting proteases that do not require an extended recognition peptide sequence beyond the primed site (i.e., no additional amino acids after the cleaved residue on the C-terminal end).

**Figure 2:**
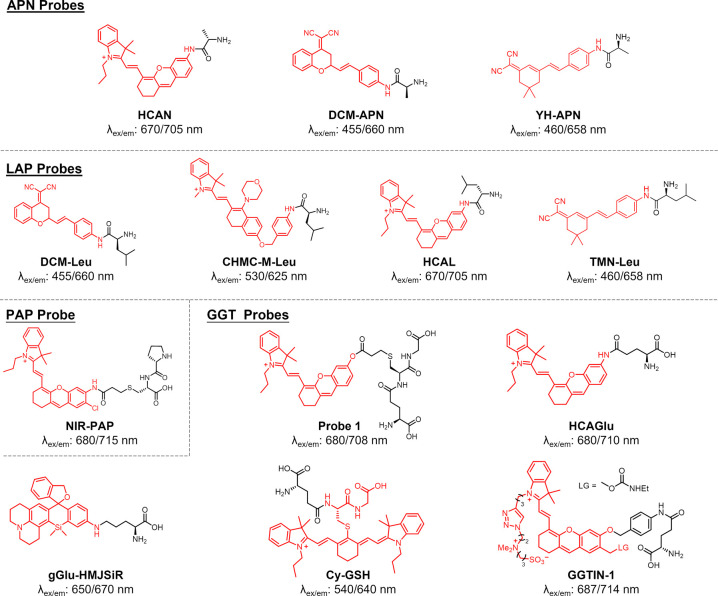
Aminopeptidase NIR probes. Representative ICT-based NIR fluorescent “turn-on” probes for imaging the activity of leucine aminopeptidase (LAP), aminopeptidase N (APN), proline aminopeptidase (PAP), and γ-glutamyltranspeptidase. Reporter (red) and targeting moiety (black) are shown with the absorbance/emission wavelengths upon enzyme activation.

Aminopeptidase N (APN) is regarded as an important biomarker for cancer as it is highly expressed in many malignant tumors.^28^ APN is mainly found in the liver, small intestine, and kidneys and plays an important role in cytokine processing, antigen presentation, and cell motility. Furthermore, the proteolytic activity of APN is believed to facilitate tumor metastasis, and its activity may be indicative of the invasiveness of cancer cells.^29^ Given that the ectodomain of APN contains a zinc-dependent active site that can preferably hydrolyze N-terminal alanyl residues,^30^ Ma et al. developed HCAN (Figure 2) as the first NIR fluorescent probe for APN, based on a hemicyanine dye conjugated to an alanyl residue.^31^ The amide bond formed between the dye and amino acid residue induces an electron-withdrawing effect on the lone pair of the aniline core, which quenches the fluorescence emission of the fluorophore. Upon cleavage of the alanine residue by APN, a 23-fold fluorescence enhancement was observed at 705 nm. HCAN showed a limit of detection for APN of 0.8 ng mL^–1^ and was able to detect its enzymatic activity in vivo in tumor-bearing mice.

More recently, Peng et al. developed a NIR fluorescent probe for the detection of APN using two-photon microscopy.^32^ In this work, the authors conjugated dicyanomethylene benzopyran (DCM) to an alanine residue to furnish the silent probe DCM-APN (Figure 2). Upon enzymatic cleavage by APN, the probe underwent a large increase in fluorescence at 664 nm with a remarkable Stokes shift of 194 nm. Furthermore, DCM-APN was able to detect APN activity between healthy and cancerous tissues and in vivo in mice bearing HepG-2 xenograft tumors. In 2020, the same group improved the detection of APN through the development of YH-APN.^33^ A similar strategy to the one used for DCM-APN was applied, but in this case the alanine residue was conjugated to a dicyanoisophorone fluorophore to furnish the probe YH-APN. YH-APN demonstrated enhanced kinetics over previous probes and achieved high fluorescence signals in primary and metastatic tumors in vivo, which highlights its potential as a chemical tool for tumor resection and metastasis detection.

Leucine aminopeptidase (LAP) is another aminopeptidase that holds potential as a cancer-imaging biomarker, given that LAP activity has been positively correlated to the progression of breast cancer and ovarian cancer, among others.^34^ Therefore, chemical tools to determine LAP activity in living systems may provide clinicians with useful information to diagnose, treat, and manage cancer patients more effectively. Similar to APN, the enzyme LAP hydrolyzes N-terminal amino acid residues, specifically leucine. A series of substrate-based NIR fluorescent probes have been designed to detect the enzymatic activity of LAP. In 2016, Zhu et al. reported a NIR fluorescent probe using DCM as the fluorophore and l-leucine as the recognition unit to generate DCM-Leu (Figure 2).^35^ The addition of leucine to the DCM scaffold altered the ICT and resulted in a weak fluorescence at 535 nm. When the leucine residue was cleaved by LAP, strong fluorescence was observed at 660 nm due to the restoration of ICT. Zhu et al. utilized the change in fluorescence from 535 nm (prior to LAP mediate cleavage) to 660 nm to establish DCM-Leu as a ratiometric probe as one of the first examples of quantification of intracellular LAP activity in live cells. To overcome the water insolubility issues of some NIR fluorophores, Wang et al. developed a more water-soluble LAP probe (CHMC-M-Leu) by introducing a morpholine moiety on the fluorescent reporter and to improve reactivity a *p*-aminobenzyl alcohol (PABA) as a self-immolative linker between the fluorophore and leucine (Figure 2).^36^ Upon the addition of LAP, great enhancement of fluorescence was observed at 625 nm due to substrate cleavage, and this probe was successfully employed to visualize LAP activity in HeLa cells.

Ma and co-workers reported one of the first NIR fluorescent probes for in vivo imaging of LAP in 2017.^37^ By conjugating a leucine residue to a hemicyanine dye, the HCAL probe (Figure 2) exhibited a strong “turn-on” fluorescence signal at 705 nm with a 32-fold enhancement over the control. HCAL was utilized to detect endogenous LAP in vivo in a drug-induced liver injury model and in xenograft HepG2 tumor-bearing mice as examples of its translational potential. In a similar approach, Kong et al. reported TMN-Leu as probe where a leucine residue was coupled to a dicyanoisophorone reporter to produce a large increase in emission with LAP activity at 658 nm.^38^ TMN-Leu showed a detection limit of 0.38 ng mL^–1^ and was described to image LAP activity in vivo in HCT116 tumor-bearing mice.

Prolyl aminopeptidase (PAP) is a type II integral membrane protein, which cleaves proline residues and proline-containing dipeptides from the terminal sequence of proteins. It is widely expressed in epithelial and nonepithelial tissues and features prominently in type 2 diabetes. In cancer, its activity has been reported to correlate inversely to the progression of carcinoma and melanoma; however, the expression of PAP as a receptor for tumor-associated fibronectin on endothelial cells has been shown to promote tumor cell adhesion and metastasis.^39^ NIR-PAP (Figure 2) was the first NIR probe for monitoring PAP activity.^40^ The probe consists of an acryloylated fluorophore Ac–Hcy–OH and a cysteine-proline dipeptide. The acylation of the hydroxyl moiety of the dye quenched the probe initially due to ICT. When the proline residue was cleaved by PAP, the probe underwent a spontaneous intramolecular cyclization releasing the Hcy–OH reporter, resulting in a large fluorescence enhancement at 715 nm with an associated detection limit of 0.013 U mL^–1^. NIR-PAP was utilized in microscopy and flow cytometry experiments to monitor PAP activity across different cell lines, with the strongest activity being detected in cancerous HepG2 cells, which highlights its use as potential biomarker of cancerous cells alongside other aminopeptidases.

γ-Glutamyltranspeptidase (GGT) is an ectoenzyme that plays a critical role in regulating glutathione homeostasis.^41^ By catalyzing the hydrolysis of the γ-glutamate group of glutathione, cysteinyl-glycine—a crucial carbon source for cancer cells—is generated. Overexpression of GGT has been found within several types of tumor cells, especially in liver cancers,^42^ and technologies capable of detecting its activity in vivo may offer significant translational potential for the diagnosis of cancer. Building on prior work from Urano and colleagues with GGT-responsive green fluorophores,^43^ the first NIR fluorescent probe for imaging GGT activity was reported by Ma et al.^44^ Probe **1** (Figure 2) was designed by attaching the hemicyanine fluorophore (*E*)-2-(2-(6-hydroxy-2,3-dihydro-1H-xanthen-4-yl)vinyl)-3,3-dimethyl-1-propyl-3H-indol-1-ium iodide (HXPI) to the recognition unit glutathione through an acrylyl linker, which resulted in good water solubility compared to previous non-NIR fluorescent probes. When probe **1** was activated by GGT, the HXPI fluorophore was released through intramolecular cyclization, and fluorescence was released with emission around 708 nm. Probe **1** was then utilized to image the activity of GGT in vivo in zebrafish. Improvements of this chemical design have been reported by the same group because the first strategy involved tandem self-immolative reactions that slowed down the detection of fluorescence response.^45^ The hydroxyl hemicyanine fluorophore HXPI was replaced by an aniline hemicyanine fluorophore HCA, which was directly conjugated to glutathione, to observe a rapid “turn-on” response at 710 nm after reaction with GGT. This probe, HCAGlu (Figure 2), was then used to visualize GGT activity in vivo in mice bearing HepG2 tumors. Other examples with different NIR fluorophores attached to the γ-glutamate group have been reported in recent years. The silicon-based rhodamine NIR fluorophore was utilized by the group of Urano to generate the probe gGlu-HMJSiR (Figure 2), which showed excellent signal enhancement (over 700-fold) at 660 nm after being activated by GGT.^46^ Peng and co-workers used the dicyanoisophorone fluorophore to demonstrate its application potential in tumor diagnosis and removal.^47^ More recent work by Li and Ye has been focused on targeting GGT activity in vivo with ratiometric probes.^48,49^ The group of Yang reported Cy-GSH as a probe including a cyanine reporter showing a hypsochromic shift in emission from 805 to 640 nm following enzymatic cleavage.^49^ This minimal crosstalk due to the large Stokes shifts^50^ could avoid signal interference between the dye pair and successfully detected small cancerous lesions in colon cancer. Meanwhile, Xie and co-workers recently developed the self-immobilizing NIR probe GGTIN-1, which undergoes enzymatic glutamyl cleavage followed by self-immolative elimination of a PABA linker and finally covalent conjugation to either GGT or nearby proteins via an exposed electrophilic trap.^51^ GGTIN-1 was administered intravenously in mice to successfully image GGT activity in tumors from up to 1 h post injection and up to 24 h in total.

### Serine Proteases

Serine proteases make up approximately one-third of human proteases and are responsible for multiple physiological processes, from digestion to blood coagulation.^52^ The dysregulation of serine protease activity has been correlated to tumor growth, survival, and metastasis,^53^ therefore the detection of serine proteases holds promise to guide clinicians for improved cancer diagnosis and personalized treatment.^54^

The catalytic capabilities of serine proteases have prompted the design of different activatable fluorescent probes for imaging their role in cancer. Fibroblast activation protein-alpha (FAPα) is a serine protease that has been recently associated with epithelial cancers, including ovarian cancer.^55^ FAPα is commonly expressed in fibroblasts of malignant tumors while absent from normal tissues.^56^ Interestingly, FAPα demonstrates exopeptidase activity (i.e., the ability to cleave terminal residues) and endopeptidase activity (i.e., ability to cleave amide bonds within a peptide sequence), which has been exploited in the chemical design of activatable probes.^57^ In 2012, Cheng et al. developed one of the first NIR fluorescent probes for FAP (ANP_FAP_) for imaging FAPα activity using a fluorophore:quencher Forster Resonance Energy Transfer (FRET) pair containing a Cy5.5 dye and a QSY21 quencher.^58^ The FAPα targeting moiety consisted of an octapeptide of sequence KGPGPNQC, which could be cleaved by FAPα between the glycine and proline residues and render increased fluorescence signals in areas of high FAPα activity. ANP_FAP_ was utilized to image FAPα activity in vivo in murine models of cancer with high tumor uptake and showed excellent signals 2 h after tail vein injection. More recently, Pu et al. developed another NIR probe (FNP1) for imaging FAPα activity in fibrotic keloid cells.^59^ The probe was comprised of a hemicyanine dye conjugated to the peptide substrate Cbz-Gly-Pro via a self-immolative PABA linker, which rendered the probe silent until FAPα cleaved the bond between the PABA linker and the proline residue to release the hemicyanine scaffold with a 45-fold fluorescence increase at 710 nm. FNP1 was employed to selectively image FAPα activity in keloid cells in human epidermal tissue with a low limit of detection of 20 000 cells. Wu et al. targeted FAPα activity in a similar manner by coupling the same hemicyanine dye directly to a Ac-Gly-Pro peptide substrate without a spacer group.^60^ The resulting HCFP probe showed a large fluorescence enhancement upon incubation with FAPα and was successfully administered intratumorally to image FAPα activity in vivo in mice grafted with MCF-7 tumors.

Neutrophil elastase (NE) is another major serine protease with a key role in the human immune system. It is expressed in neutrophils, the most abundant white blood cells in the body, which are part of the innate immune system involved in antimicrobial defense and inflammation. More recently, NE has been shown to have a pro-tumorigenic role in several types of cancer.^61^ One of the first NIR probes for measuring NE activity (NE680) was reported in 2011.^62^ The chemical design of NE680 included two NIR-emitting fluorophores (VivoTagS-680) that were coupled together via a nonapeptide (PMAVVQSVP) sequence functionalized with an amphiphilic polymer. In the absence of active NE, the NE680 probe was silent due to ground state static quenching of the two fluorophores. However, upon interaction with the enzyme, the cleavage of the peptide sequence resulted in a 30-fold fluorescence increase around 680 nm, and the probe was successfully applied in vivo in mouse models of lung injury. In addition to some advances in the design of non-NIR fluorescent peptide-based probes,^63^ Yang et al. reported a NIR probe building on the discovery of a nonpeptidic scaffold for human NE.^64^ Specifically, the authors developed the probe NEP by incorporating a pentafluoropropanoyl moiety to a hemicyanine dye, which was quenched through ICT. A 25-fold fluorescence increase at 700 nm was achieved after the reaction between NEP and active human NE with a limit of detection of around 30 ng mL^–1^. NEP demonstrated its ability to visualize NE trafficking in vitro and importantly was able to image NE activity in mouse models. More recently, the use of the pentafluoroethyl ketone as a targeting group for NE has furnished two additional activatable NIR probes using a similar ICT-based quenching system to detect NE activity in live cells.^65,66^ Drag and colleagues developed a suite of ABPs (ranging from green to NIR) for monitoring the localization of serine proteases in neutrophil azurophil granules.^67^ They developed diphenyl phosphonate covalent inhibitors featuring unnatural amino acids not only for NE but also for cathepsin G, proteinase 3, and neutrophil proteinase 4. Simultaneous imaging of all four serine proteases in vitro in human neutrophils revealed a hitherto discriminatory packing system of serine proteases in azurophil vesicles.

**Figure 3:**
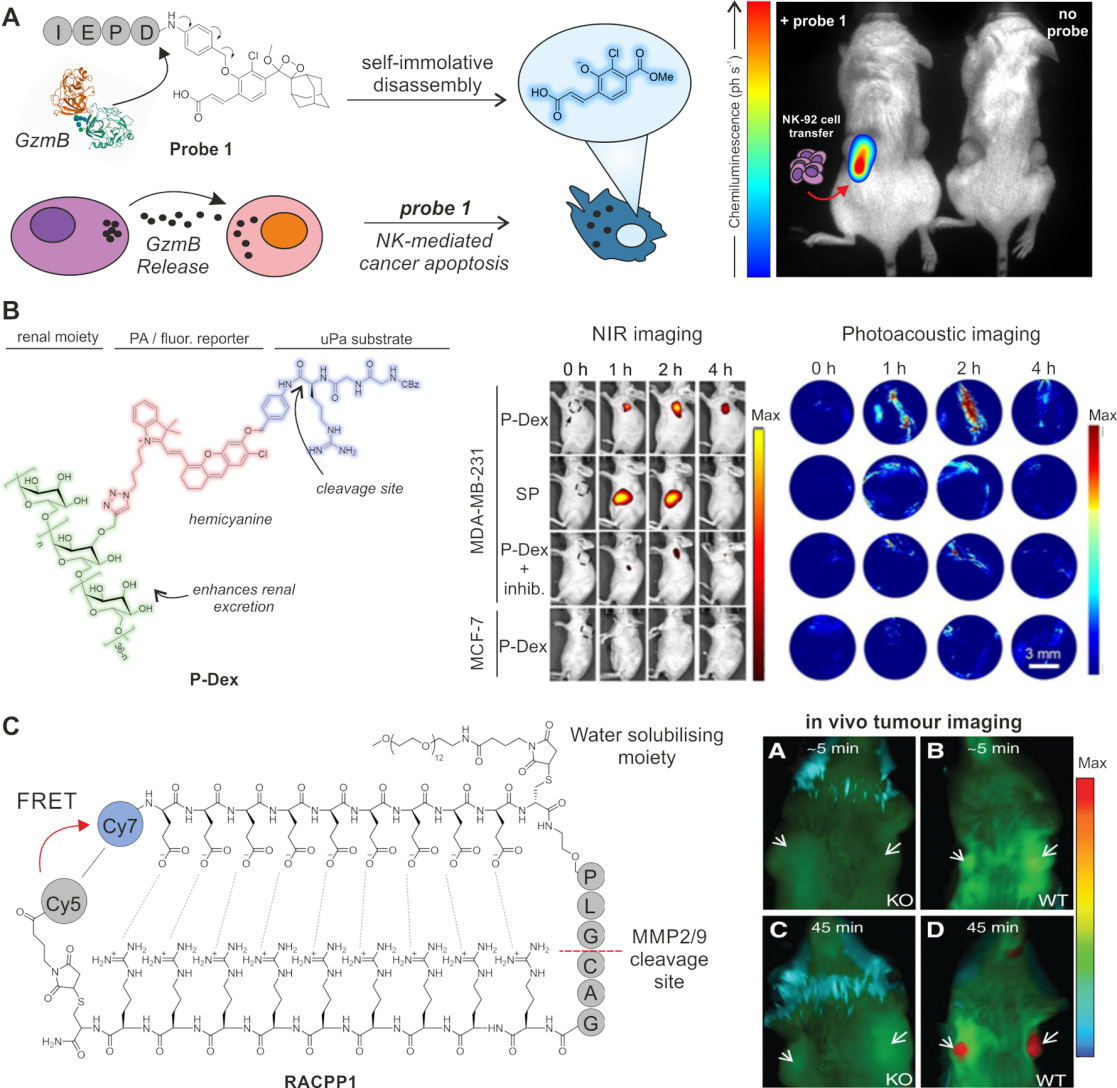
In vivo imaging activatable probes for serine proteases. (A) Chemical structure and mechanism of probe **1** with active GzmB and whole-body imaging of tumor-bearing mice. (Left mouse) Only the right tumor (red arrow) was injected with NK92 cells with the left tumor being NK cell-free. After 8 h of NK cell injection, probe **1** was injected into both tumors. (Right mouse) Control animal without probe **1**. Adapted with permission from ref (74). Copyright Wiley-VCH 2021. (B) Chemical structure of P-Dex and NIR and PA of uPA-expressing tumors. NIR fluorescence images of tumor-bearing mice after administration of P-Dex with and without uPA inhibitors. PA images of tumors after systemic treatment with P-Dex as outlined in fluorescence images. Adapted with permission from ref (78). Copyright 2020 Wiley-VCH. (C) Chemical structure of RACPP1 and images of mice bearing tumors with wild-type MMP (WT) or knockout MMP (KO). Pseudocolor red indicates cleaved probe, green indicates intact probe, and arrows indicate tumor sites. Adapted with permission from ref (87). Copyright 2013 American Association for Cancer Research.

In addition to biomarkers that are overexpressed in cancer cells, recent efforts have focused on evaluating biomarkers that can report the immune response against cancer, like the serine protease granzyme B (GzmB). GzmB is a serine protease stored within the cytotoxic granules of T cells and natural killer (NK) cells, which is released upon engagement with cancer cells and responsible for initiating apoptosis.^68^ Seminal work was performed in evaluating the specificity of tetrapeptide substrates for GzmB by Thornberry et al. in 1997.^69^ The optimum sequence identified was Ile-Glu-Pro-Asp, and this has been used successfully to target GzmB in a range of ways (e.g., inhibitors, PET imaging agents, and optical reporters).^70−73^ Recently, our group developed the first chemiluminescence probe for in vivo imaging of GzmB activity released by NK cells utilizing the IEPD tetrapeptide sequence coupled to a dioxetane chemiluminescence reporter.^74^ When incubated with GzmB, probe **1** underwent a 139-fold increase in chemiluminescence and was able to specifically image GzmB activity in vivo in NK cell treated tumors in mice (Figure 3A). Previously, Kwong et al. had developed a series of GzmB reactive nanosensors for assessing acute transplant rejection in mice.^75^ In this case, IRDye-800CW was coupled to the kGGsIEFDSGGGs[PRA]c peptide via a propargylglycine (PRA), while a NIR quencher—structure not disclosed—was attached to the N-terminus. The probe achieved a 4-fold increase when incubated with recombinant GzmB and was able to successfully detect GzmB activity in early stages of allograft rejection *ex vivo*. Meanwhile Kulkarni et al. recently synthesized a GzmB nanoreporter for monitoring tumor response to immunotherapy.^76^ The nanoreporter contained a GzmB-cleavable substrate (GKIEPDAPC) with a DyLight755 reporter attached to the lysine side chain and a DyLight766 quencher conjugated to the cysteine thiol group. The nanoreporter was also modified with a PD-L1 blocking antibody to boost the T cell immune response and subsequently increase release of active GzmB. αPDL1-GNR successfully imaged GzmB activity in vivo in tumors of mice with higher levels observed in the antibody fused nanoreporter vs the control. Some of the first NIR fluorescent probes for GzmB were developed by Pu et al. and featured a PEGylated hemicyanine dye conjugated to the GzmB-responsive tetrapeptide sequences for mouse (CyGB_F_: IEFD sequence) or human (CyGB_P_: IEPD sequence) enzymes.^77^ Upon interaction with the active enzymes, the probes were cleaved and furnished fluorescence increases around 25-fold for CyGB_F_ and 22-fold for CyGB_P_. The authors used both probes in tumor-bearing mice treated with immunomodulatory drug compounds and were able to detect increased GzmB activity in the tumors of treated mice. Owing to their high renal clearance, both probes were also used for urinalysis. The same group recently developed a dual NIR-photoacoustic (PA) probe for monitoring the activity of urokinase-type plasminogen activator (uPA), a serine protease that has been implicated in breast cancer and metastasis formation.^78^ In this case, a glycosylated hemicyanine dye was conjugated to the uPA-specific peptide sequence Cbz-Gly-Gly-Arg via a PABA linker to furnish P-Dex. A renal clearance moiety was included in the probe as PA agents suffer from poor clearance from the body and may prevent clinical translation. Upon cleavage, the probe rendered a 13-fold increase in fluorescence intensity at 725 nm and a 2-fold increase in the PA readout at 690 nm. P-Dex was successfully used to distinguish between cancerous tissue overexpressing uPA and healthy tissues in vivo in murine models (Figure 3B).

### Matrix Metalloproteases (MMPs)

Another class of proteases that has received significant attention in relation to cancer imaging are MMPs. These proteases are involved in remodelling the extracellular matrix, and they have been reported to promote tumor cell invasion and angiogenesis.^79^ Classical FRET approaches to target MMP activity with NIR fluorophore:quencher pairs have been described over the past two decades.^80−82^ In 2012, Nagano et al. sought to address limitations of cell permeability and toxicity of MMP probes by developing a series of intracellularly retained NIR activatable probes following their cleavage by MMP-2, MMP-9, or membrane-type 1 MMP (MT1-MMP).^83^ These probes featured a NIR dye (e.g., sulfo-Cy5, Cy5, BODIPY, or Si-Rhodamine) coupled to a BHQ-3 dark quencher via a MMP-cleavable peptide sequence (PLGLAG). Cell-based studies revealed that the C-terminal BODIPY probe BODIPY-MMP had optimal cell permeability and was successfully used to image MMP activity in vivo in a mouse xenograft model. Chen et al. also developed a NIR FRET-based probe for monitoring the activity of MMPs in vivo utilizing a Cy5.5 dye and a BHQ-3 quencher tethered by the pan-reactive MMP substrate GPLGVRGKGG.^84^ However, the probe suffered from nonspecific cleavage by other enzymes. To improve the MMP selectivity, the authors conducted a metabolic analysis and identified that the replacement of l-lysine by d-lysine reduced nonspecific cleavage and improved the tumor-to-background ratios of initial constructs when employed for in vivo imaging.^85^ Interestingly, these probes have also been used to image MMP-9 expression in vivo in mouse models of diabetic stroke, demonstrating potential clinical utility across several diseases.^86^

In other approaches, Nguyen and co-workers developed ratiometric probes for imaging of MMP-2 and -9 as well as elastase activity.^87^ The probe RACPP1 included a cell-penetrating poly-d-arginine sequence that was conjugated to a Cy5 dye and a poly-d-glutamate sequence linked to a Cy7 dye. Owing to the strong ionic interaction between the two peptides, they remained bound to each other and unable to enter cells until MMP cleavage released the poly-d-glutamate sequence and the Cy5-labeled fragment could enter cells showing around 40-fold increased fluorescence signals. The ratiometric signal of RACPP1 enabled the successful detection of primary tumors and metastases to the lymph nodes and liver in mice (Figure 3C). In a phase 1b clinical trial (NCT02391194), RACPP1(or AVB-620) generated no adverse events and was able to discriminate between tumor tissue and healthy tissue in women with breast cancer.

Probes for dual-modality imaging (i.e., NIR fluorescence and either PET/SPECT or PA) have also been reported for the detection of MMP activity in the context of cancer. Gao and colleagues described cRGD-QC as an MMP-2 activatable probe featuring a Cy5 dye, a QSY21 quencher, and a tumor targeting cyclic RGD covalently bound through a ^125^I labeled peptide substrate.^88^ Upon cleavage by active MMP2, cRGD-QC emitted a strong NIR signal and the cRGD tumor-targeting moiety enhanced probe *in vivo* accumulation in tumors, which was detected by NIR fluorescence and SPECT imaging. Interestingly, Gao then developed a NIR/PA probe for interrogating MMP-2 activity *in vivo* in mice.^89^ The probe, QC, comprises a Cy5.5 dye linked to a QSY21 quencher via a MMP-2 cleavable substrate (GPLGVRGY). Upon cleavage, an increase in NIR fluorescence was detected as well as a drop in PA signal at 680 nm (meanwhile, PA internal reference signal at 730 nm stayed constant) allowing for successful dual imaging of breast cancer in murine models. More recently, Warram et al. developed a NIR and PET-based dual imaging agent for MMP-14 activity by utilizing the IRDye800 reporter and IR-QC-1 dark quencher linked to an MMP-cleavable substrate modified with a NOTA chelator for radioisotopes (e.g., ^64^Cu).^90^ This construct releases NIR emission in areas of high MMP-14 activity and can bind to the membrane-bound protease. Notably, the NIR fluorescence signals correlated well with the PET signals *in vivo* and colocalized with the expression of MMP-14 in human glioma orthotopic xenografts. Xu et al. developed a novel self-assembling NIR nanofiber imaging probe for the detection of MMP2 and MMP9 activity in renal cell carcinoma.^91^ The probe TER-SA consisted of a NIR cyanine dye conjugated to a large peptide, including (1) a tumor-targeting RGD sequence, (2) a PLGYLG MMP-2/9 cleavable sequence, and (3) a YLGFFC self-assembling motif. Upon MMP-mediated cleavage, TER-SA underwent self-assembly into β barrel-like structures, which resulted in strong NIR fluorescence emission. Importantly, the formation of nanofibers overcame one of the main challenges when imaging renal cancers, such as the rapid clearance of the contrast agents from the kidneys. This delay in kidney excretion allowed the detection of small lesions (<1 mm) in murine xenograft models and, remarkably, in a human kidney *ex vivo* perfusion model.

### Cysteine Proteases

One of the most heavily researched enzyme families in the human proteome are caspases. There are 12 members of the caspase family, termed caspase-1 to caspase-12, and their name is derived from their cysteine protease activity (i.e., a cysteine residue in the active site cleaves target proteins, typically after an aspartic acid residue).^92^ They have essential functions in programmed cell death, also known as apoptosis, and their activity can be either tumor-suppressing or tumor-promoting depending on the tissue microenvironment, making them interesting biomarkers in cancer research.^93^

**Figure 4:**
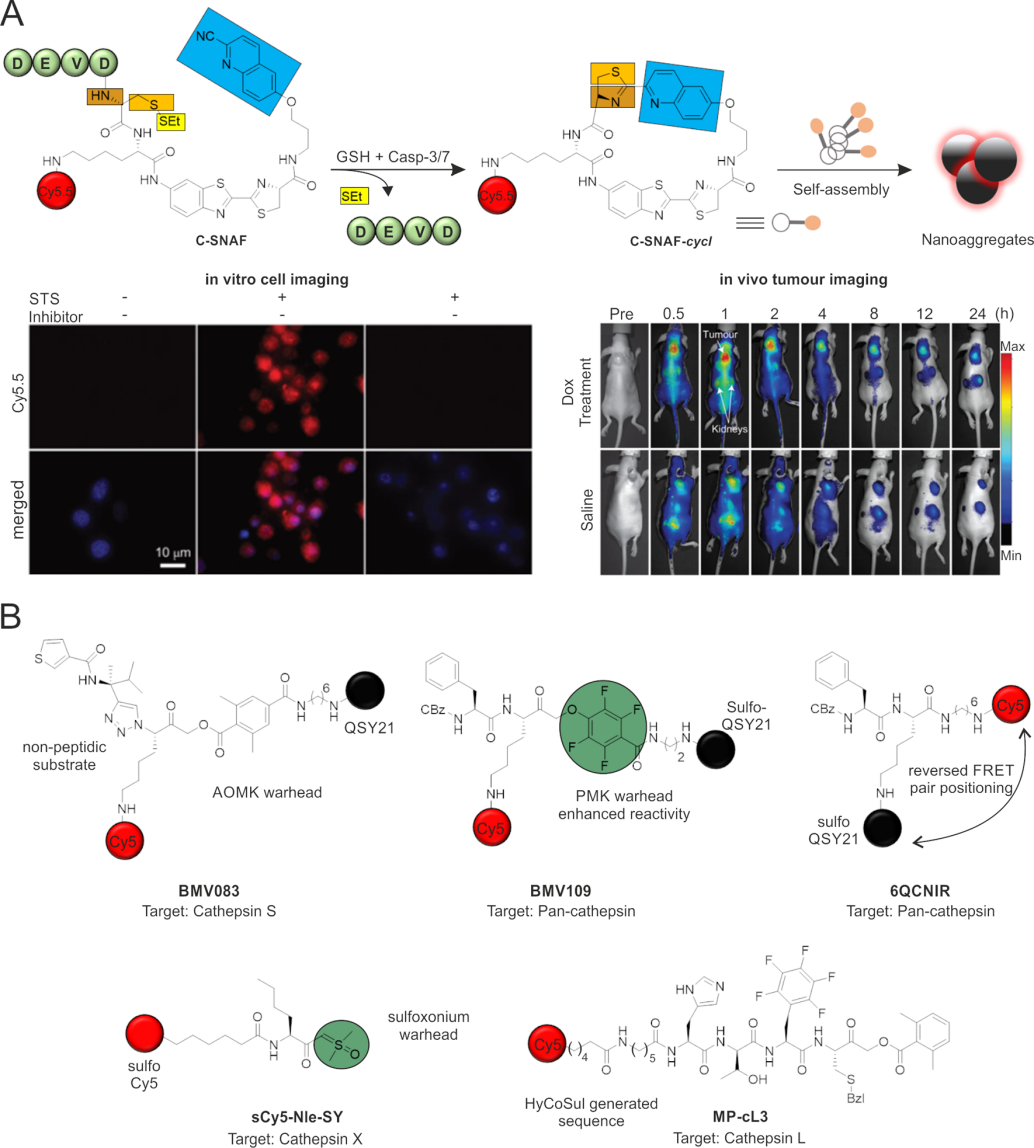
NIR activatable probes for cysteine proteases. (A) Chemical structure and aggregation mechanism of C-SNAF following reaction with caspase-3/7 and glutathione. Fluorescence microscopy images of STS-induced apoptotic HeLa cells labeled with C-SNAF, with cells stained by the nuclear dye Hoechst 33342 (blue) and fluorescence (red) in the apoptotic cells, which indicates specific intracellular accumulation of C-SNAF after caspase-3/7-triggered macrocyclization and nanoaggregation. *In vivo* longitudinal fluorescence images of HeLa tumor-bearing mice after treatment with C-SNAF and doxorubicin (DOX) or saline. Tumor and kidneys are indicated by white arrows. Mice that carry subcutaneous HeLa tumors received either 8 mg kg^–1^ DOX or saline and were imaged using a Maestro fluorescence imager. Adapted with permission from ref (97). Copyright 2014 Springer Nature. (B) Representative chemical structures of cathepsin-targeting activatable probes.

Prior to the past decade, the generation of NIR activity-based probes for imaging caspases laid strong foundations for further developments seen over the past 10 years.^94−96^ In 2014, Rao et al. developed C-SNAF as an in situ self-aggregating NIR smart probe for detecting caspase-3 and caspase-7 activity *in vivo* (Figure 4A).^97^ The probe C-SNAF contained a Cy5.5 dye flanked by the DEVD caspase-3/7 cleavable sequence and a cysteine residue modified with a disulfide bond. In tumor cells responding to therapy, the DEVD sequence of C-SNAF was cleaved by active caspases, and the resulting intramolecular cyclization led to the formation of nanoaggregates with strong NIR fluorescence signals. The probe was successfully used to image caspase activity *in vivo* in tumor-bearing mice following treatment with doxorubicin. Later on, Zhang et al. developed Mc-Probe as a multi-FRET ratiometric probe to detect the activity of caspase-3 and MMP-2 enzymes, both of which are involved in cancer progression.^98^ Mc-Probe consisted of a fluorescein unit (FAM) linked to the caspase-3 cleavage sequence DEVD and rhodamine (TAMRA) reporter after the aspartic acid cleavage site. The C-terminal end also contained an MMP-2 cleavage sequence with a terminal Dabcyl quencher. Due to the multiple FRET pairs within the construct, the dyes were initially nonfluorescent; however, caspase-3 activation released the emission of FAM, and MMP-2 activation resulted in the emission of TAMRA. If both proteases were active, the signals of TAMRA and FAM were combined in a unique ratiometric signal for spatiotemporal imaging of MMP-2 and caspase-3 activity in live cells.

More recently, the group of Pu reported the probe CFR as a dual fluorescent and chemiluminescent smart probe for imaging of liver hepatotoxicity.^99^ This construct consisted of a hemicyanine dye modified with a DEVD caspase-cleavable peptide sequence and a chemiluminescent dioxetane with a superoxide-sensing moiety for combined monitoring of apoptosis and cellular oxidative stress. Upon activation by both events, the probe CFR underwent a 12-fold increase in fluorescence at 710 nm and 40,000-fold increase in chemiluminescence emission. The authors used a mouse model of drug-induced hepatotoxicity to detect superoxide *in vivo* and caspase activity with 1.5-fold NIR fluorescence increase after treatment with valproic acid, a known inducer of hepatotoxicity. In a similar strategy, the same group developed hemicyanine-DEVD activatable probes for the imaging of drug-induced acute kidney injury,^100^ where MRP3 probes featured a 2-hydroxypropyl-β-cyclodextrin moiety for monitoring of cellular apoptosis *in vivo* in the kidneys of mice treated with cisplatin. Zhao and co-workers have recently developed Ac-Tat-DEVD-CV as a NIR ratiometric probe for imaging caspase-3 activity in live cells.^101^ Ac-Tat-DEVD-CV is a fluorogenic probe with a cresyl violet reporter anchored to the C-terminus of the caspase-specific substrate and a cell-penetrating peptide on the N-terminus. Upon caspase-mediated cleavage, the probe red-shifts its fluorescence emission and can be used to monitor caspase activity in apoptotic HeLa cells.

Compared to caspase-3 and caspase-7, much less work has been done on activatable probes for caspase-1. This enzyme plays a crucial role in initiating the inflammasome activation pathway, the dysregulation of which can lead to hyperinflammatory states associated with cancer or neurodegenerative disease. In 2020, Kwon et al. developed one of the first NIR FRET activity-based probes for imaging caspase-1 *in vivo*.^102^ Cas-1 consisted of a Cy5.5 dye attached to the N-terminus of a caspase-1-specific peptide sequence (GWEHD*GK) and a BHQ-3 dark quencher conjugated to an C-terminal lysine so that, upon cleavage of the sequence by caspase-1, a large increase in fluorescence at 710 nm could be observed. Cas-1 was highly specific for caspase-1 over other caspases and used to image *in vivo* activity in murine models of inflammatory bowel disease, colon cancer, and Alzheimer’s disease.

Another important family of cysteine proteases are cathepsins. These proteases are most associated with their role as degradative enzymes within the lysosome, where they are responsible for protein metabolism, antigen presentation, and lysosome-mediated cell death.^103^ However, they also have extracellular roles upon lysosomal exocytosis, such as in the degradation of the extracellular matrix.^104^ The overexpression and activity of cathepsins have been reported in several types of cancer, where they can support the metabolic needs of tumors, disrupt the extracellular matrix, and promote cancer cell metastasis and invasion.^105^

The majority of NIR probes for imaging cathepsins are quenched activity-based probes (qABP).^106^ These chemical tools typically consist of four groups: (1) a fluorescent reporter, (2) a cathepsin-targeting moiety, (3) an electrophilic warhead, and (4) a dark quencher. The qABP probes are quenched through a FRET mechanism so that, in the presence of active cathepsins, the cysteine active site is covalently modified by the electrophilic warhead and the quencher is released, resulting in a strong fluorescence increase. In 2012, the team of Bogyo developed qABPs featuring a non-peptide-based recognition moiety to image cathepsin S.^107^ The probe BMV083 consisted of a triazole-based cathepsin S inhibitor conjugated to a Cy5 dye and a 2,6-dimethyl benzoic AOMK electrophilic warhead modified with a QSY21 dark quencher (Figure 4B). BMV083 showed specificity for cathepsin S and was employed to visualize activity *in vivo* in murine models of cancer. The same group later extended this work by introducing 2,3,5,6-tetrafluoro-substituted phenoxymethyl ketones (PMK) as electrophilic warheads alongside a sulfonated QSY21 dark quencher to improve pan-cathepsin labeling and *in vivo* compatibility.^108^ The resulting probe BMV109 featured enhanced water solubility and was used to image cathepsin activity within tumor-bearing mice *in vivo* with a 25-fold fluorescence increase, and notably it has been used to identify tumor-associated immune cells in human polyps (Figure 1).^109^ In a related approach, Edgington-Mitchell et al. recently synthesized a sulfoxonium ylide warhead as part of an ABP capable of monitoring cathepsin X activity with notable improvement over AOMK warheads (Figure 4B).^110^ Its straightforward synthesis, which does not require diazomethanes to generate chloromethylketones, may have advantages as the warhead-of-choice in future developments of cathepsin probes.

In a more recent attempt to improve the selectivity for cathepsin S, BMV157 was reported as a probe featuring a Cy5:sulfo-QSY21 FRET pair with the AOMK electrophilic warhead and a bulkier trans-4-methylcyclohexyl recognition substituent in the position P2.^111^ It is believed that the steric bulk increases specificity for cathepsin S over other related enzymes. BMV-157 was used in dual-color localization studies of cathepsin S and other cathepsins in live primary murine cells. Further to this work, new derivatives featuring the ICG fluorophore and a QC-1 quencher have been reported for use in surgical systems.^112^ Specifically, the probe VGT-309 was tested in a proof-of-concept study to improve the efficacy of surgical removal of cancer tissue in a mouse model of breast cancer utilizing the Spy Elite and Explorer Air NIR equipped surgical systems.

In parallel to the design of pan-reactive and cathepsin B and S probes, Drag and co-workers developed MP-cL3 as one of the first cathepsin L-specific NIR ABPs (Figure 4B).^113^ Utilizing peptide-based substrate recognition, a combinatorial library was generated to identify new substrates with high specificity for cathepsin L. This work resulted in the discovery of the His-_d_Thr-Phe(F_5_)-Cys(Bzl) sequence, which was modified with a Cy5 dye via a N-terminal 6-aminohexanoic acid spacer and a C-terminal AOMK electrophilic warhead. MP-cL3 featured a large *K*_obs_/*I* of 322 000 M^–1^ s^–1^ for cathepsin L and *K*_obs_/*I* values under 1000 M^–1^ s^–1^ for other cathepsins, including cathepsin B. Using a similar strategy, the group designed a specific ABP for cathepsin B.^114^ MP-cB-2 featured an N-terminal Cy5 dye and a C-terminal AOMK warhead linked to the Cha-Leu-Glu(OBzl)-Arg peptide sequence, which demonstrated a *K*_obs_/*I* value of 76 800 M^–1^ s^–1^ for cathepsin B and *K*_obs_/*I* values under 1000 M^–1^ s^–1^ for other cathepsins. MP-cB-2 was used to detect enzyme activity in 18 different cancer cell lines and *ex vivo* in stage IV lung cancer patient samples.

While the use of electrophilic warhead activity-based probes dominates the cathepsin imaging field, Shabat et al. developed substrate-based NIR probes for imaging cathepsins.^115^ These probes incorporated a Z-Phe-Lys cathepsin B recognition unit and either featured an internal charge transfer turn-on mechanism with a napthol QCy7 dye or a FRET pair consisting of Cy5 and a cyanine dark quencher. Both probes exhibited large NIR fluorescence increases upon incubation with cathepsin B and were successfully employed to visualize cathepsin B activity *in vivo* in 4T1 mammary adenocarcinoma tumor-bearing mice. Meanwhile, phase-1 human clinical trials have recently been conducted utilizing pan-cathepsin-activated LUM015.^116^ The probe, developed by the Brigman lab, features a QSY21 quencher attached to a pan-cathepsin reactive sequence (GGRK) with a 20-kDa PEG group and a Cy5 fluorophore. LUM015 has been utilized in clinical trials for soft-tissue sarcomas and breast cancer as well as colon, esophagus, and pancreatic cancers (NCT01626066, NCT02438358, and NCT02584244). More recently, Bogyo and co-workers then furnished 6QCNIR (Figure 4B) as a pan-cathepsin reactive NIR substrate-based probe for use in fluorescence-guided surgery.^117^ The probe 6QCNIR was developed with a NIR Cy5 fluorophore at the C-terminus and a sulfo-QSY21 at the N-terminus. The probe demonstrated its potential clinical utility via successful intraoperative fluorescence-guided detection and resection of colorectal, breast, and lung tumors in preclinical mouse models using the da Vinci surgical system.

In addition to imaging probes, NIR theranostic activatable compounds have also been reported for exploiting cathepsin activity.^118−120^ Yoon and co-workers developed the NIR fluorescent and photodynamic therapy agent CyA-P-CyB by utilizing two cyanine moieties tethered by the GPLG cathepsin B substrate. The fluorescence at 720 nm was quenched by the FRET effect between the cyanine moieties, and the photosensitizer showed cytotoxic action only when it was excited at 808 nm. Upon peptide cleavage by cathepsin B, the FRET effect was reduced, and the enzyme activity was detectable by fluorescence while the photodynamic illumination at 808 nm led to cancer cell death *in vivo* in mouse models.

## Conclusions and Outlook

The enhanced optical properties of NIR probes make them interesting scaffolds for the design of molecular imaging agents. Multiple approaches for the synthesis and validation of NIR activatable fluorescent probes in biological systems have been described over past decades. In particular, many of these chemical strategies have been focused on the optimization of NIR reporters for proteases owing to their direct links to cancer-associated events. The chemical diversity within this rapidly growing molecular toolbox is very rich, including (1) ICT-dependent activatable fluorophores and FRET fluorophore:quencher pairs as NIR-emitting systems and (2) peptides, small inhibitors, and nanoconstructs as protease-targeting agents.^121^ It is reasonable to expect that the consolidation of these synthetic methodologies will facilitate the design of more NIR probes for other enzymes (e.g., glycosyl or lipid transferases, isomerases) that are relevant to the chemical biology community. Regardless of their target, one potential obstacle in the clinical translation of NIR probes is their limited water solubility, largely due to the lipophilic nature of many NIR scaffolds. This is particularly critical in FRET probes which contain two relatively large hydrophobic structures (i.e., fluorophore and quencher). Recent work in the design of NIR activatable structures with potential compatibility for protease sensing^122^ will help to overcome the need for quenchers and potentially improve solubility profiles. The conjugation of activatable fluorophores to small soluble proteins has been also described as an effective approach to improve not only target selectivity but also the biodistribution of fluorophores *in vivo*.^14^ Furthermore, the compatibility of NIR activatable probes with several imaging modalities—from preclinical *in vivo* imaging to fluorescence-guided surgery—demonstrates their utility for multiple applications, expanding from drug discovery and mechanistic biological studies to more clinically relevant assays. Among these, the design of NIR optoacoustic probes for protease sensing is particularly interesting,^89^ given the increased penetration depth of optoacoustic imaging and several clinical studies demonstrating its use for noninvasive imaging in humans.^123^ Finally, recent advances in dual and theranostic probes^124^ will pave the way toward the design of multimodal imaging probes as well chemical agents able to deploy therapeutic loads, offering more sophisticated tools to answer complex biological questions and to accelerate precision medicine.

## Supporting Information Available

Tables listing the chemical structure, target, optical properties, and references for all of the probes discussed in the manuscript (PDF)

## Keywords

**Cancer**: A disease caused by an uncontrolled division of abnormal cells in a part of the body
**Fluorescence**: The emission of light by a chemical that has absorbed light
**Near-Infrared**: Light in the near-infrared region of the electromagnetic spectrum
**Peptide**: A short chain of amino acids
**Probe**: A molecule capable of generating a signal in response to triggering stimuli
**Protease**: An enzyme that catalyzes the hydrolysis of peptide bonds
**Quencher**: A molecule capable of absorbing the light emitted by a fluorophore
**Substrate**: A biomolecule capable of reacting and be cleaved by proteases
