# Supplementary material for: Near-Infrared Fluorescent Probes for the Detection of Cancer-Associated Proteases
Source: ACS Chem Biol. 2021 Jul 27;16(8):1304–17. doi: 10.1021/acschembio.1c00223 (PMC8383269; doi:10.1021/acschembio.1c00223)
Supplement: Supplementary file 1 — cb1c00223_si_001.pdf [file cb1c00223_si_001.pdf]

Electronic Supporting Information

Near-infrared fluorescent probes for the detection of cancer-associated proteases

Jamie I. Scott, Qinyi Deng, Marc Vendrell

Centre for Inflammation Research, The University of Edinburgh, EH16 4TJ Edinburgh, UK

Table S1. Code, chemical structure, target enzyme, absorbance/emission wavelengths and reference number of probes for aminopeptidases.

**Code**	**Structure**	**Target**	**$\lambda_{abs/em}$ (nm)**	**Ref**
HCAN	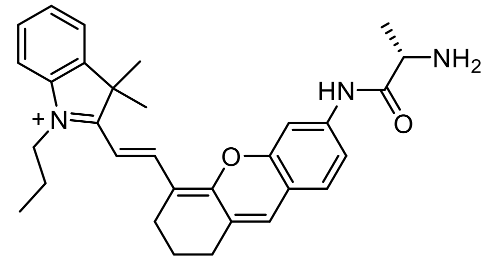	APN	670/705	31
DCM-APN	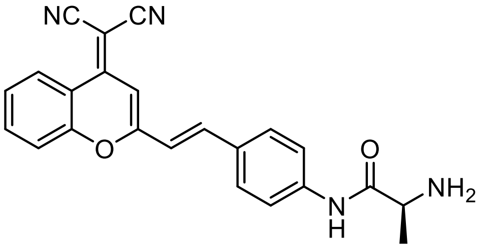	APN	455/660	32
YH-APN	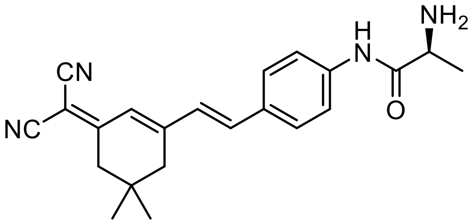	APN	460/658	33
DCM-Leu	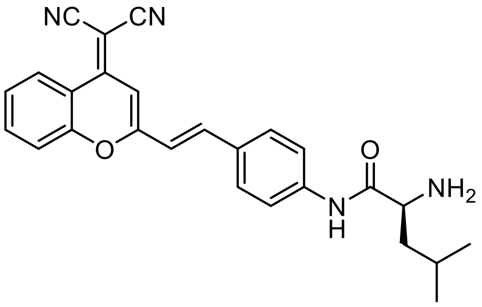	LAP	455/660	35
CHMC-M-Leu	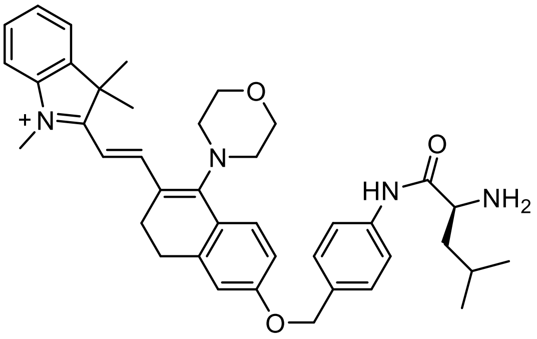	LAP	535/625	36

HCAL	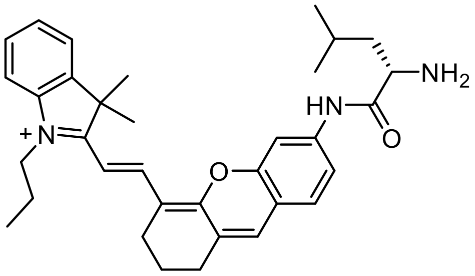	LAP	670/705	37
TMN-Leu	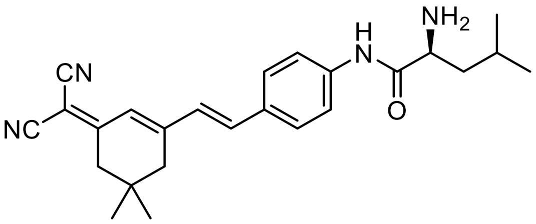	LAP	460/658	38
NIR-PAP	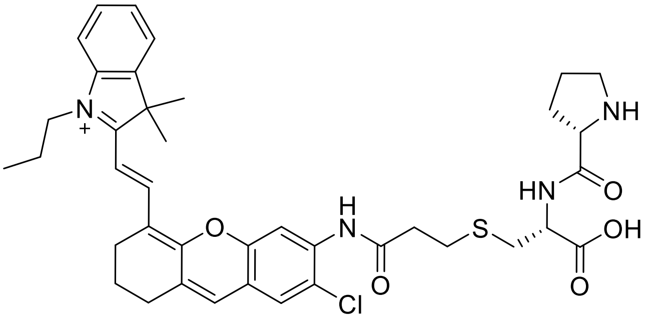	PAP	680/715	40
Probe 1	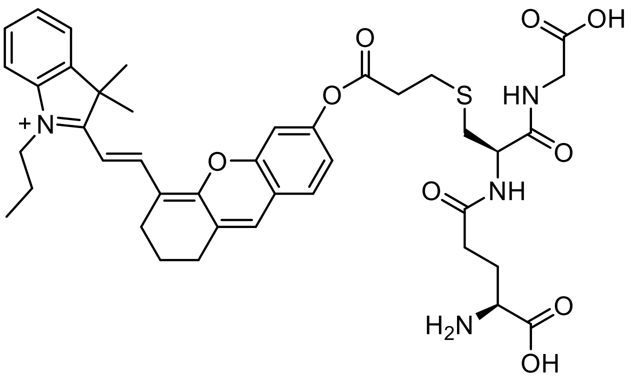	GGT	680/708	44
HCAGlu	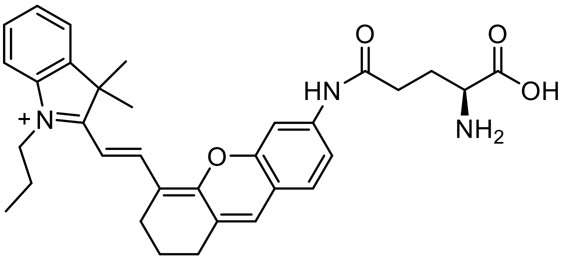	GGT	680/710	45
gGlu-HMJSiR	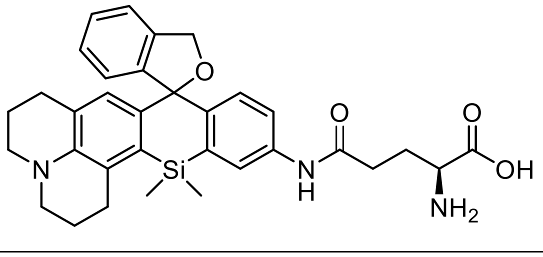	GGT	650/670	46

Cy-GSH	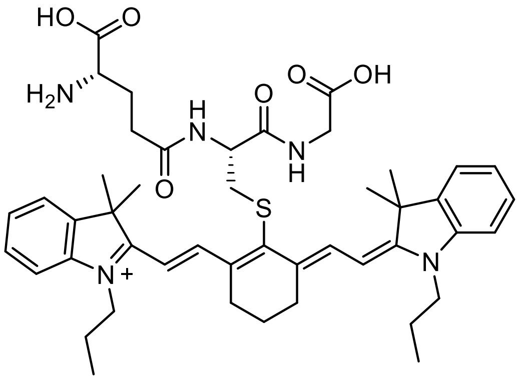	GGT	730/805	49
GGTIN-1	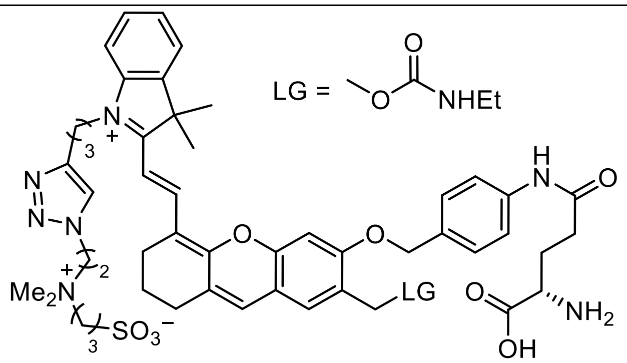	GGT	687/714	51

Table S2. Code, chemical structure, target enzyme, absorbance/emission wavelengths and reference number of probes for serine proteases.

**Code**	**Structure**	**Target**	**$\lambda_{abs/em}$ (nm)**	**Ref**
ANP$_{FAP}$	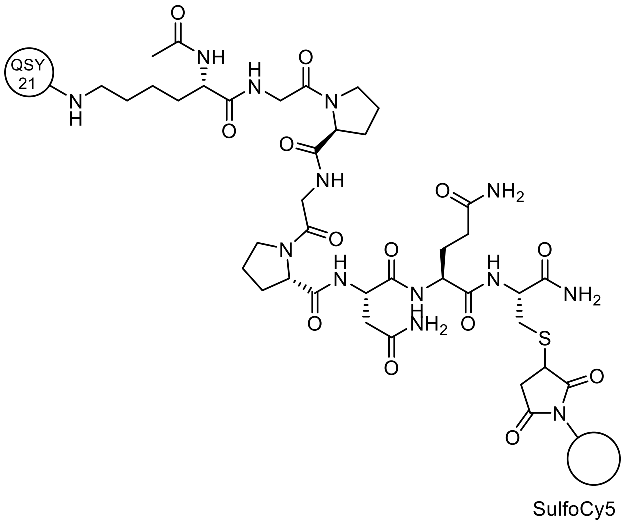	FAPα	670/695	58
FNP1	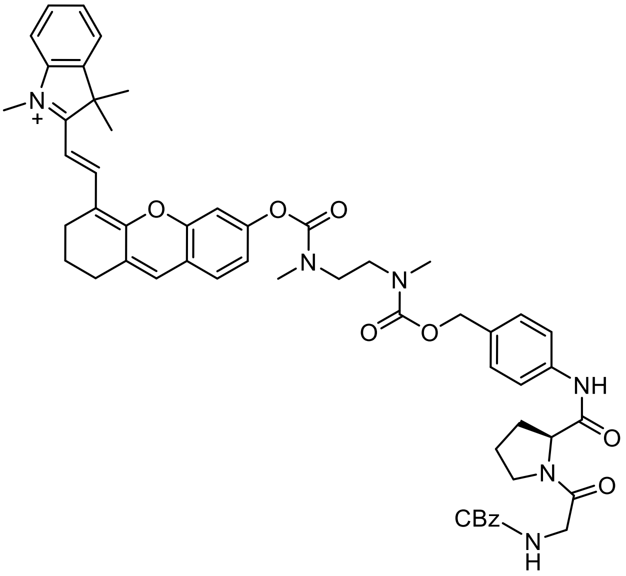	FAPα	680/715	59
HCFP	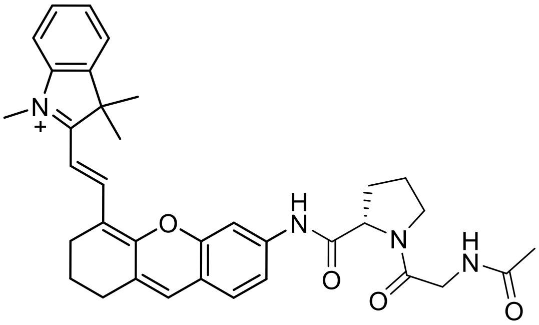	FAPα	670/710	60

NE680	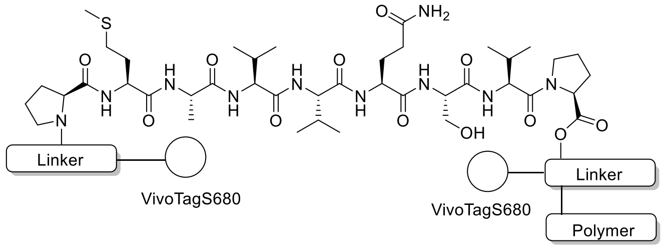	NE	680/695	62
NEP	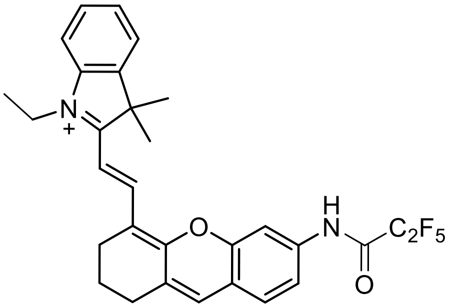	NE	680/<br>700	64
Probe 1	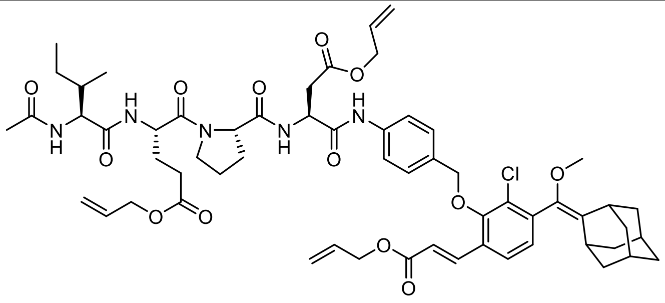	GzmB	NA / 520	74
NIR<br>GzmB	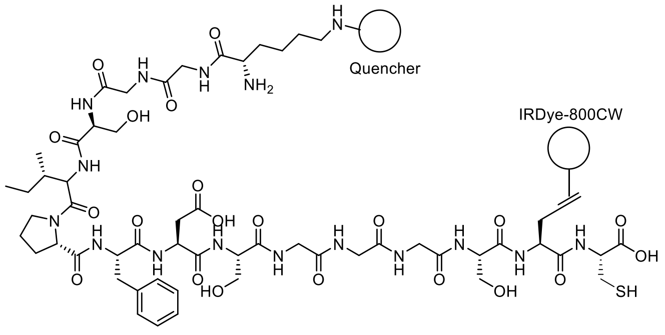	GzmB	778/794	75
αPDL1<br>-GNR	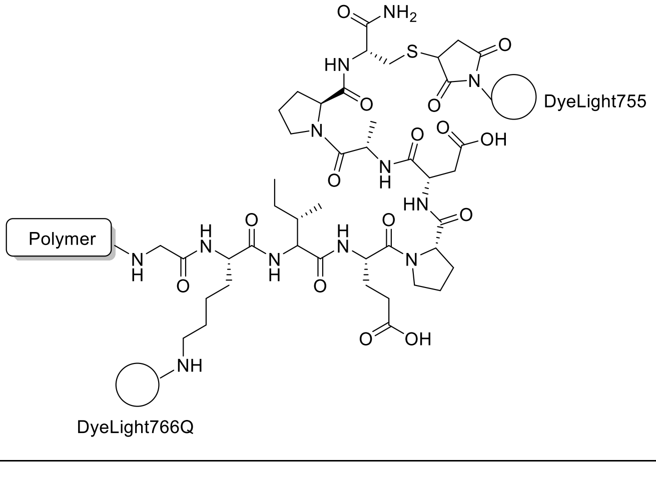	GzmB	754/776	76

$CyGB_F$	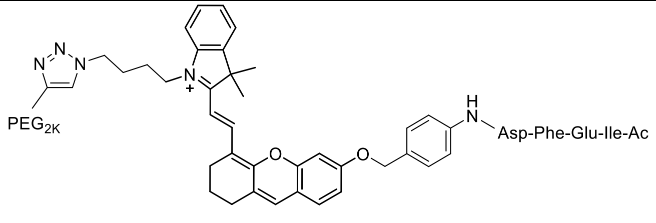	GzmB	680/710	77
P-Dex	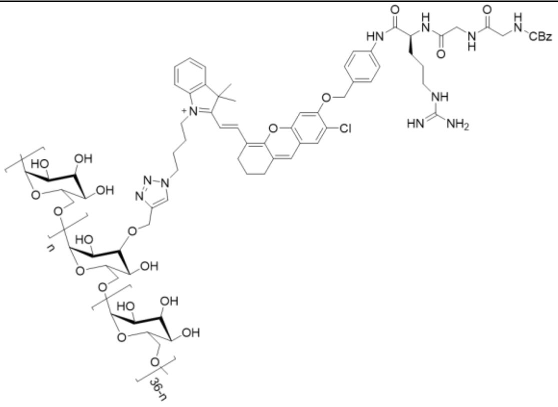	uPA	680/710	78

Table S3. Code, chemical structure, target enzyme, absorbance/emission wavelengths and reference number of probes for matrix metalloproteases.

**Code**	**Structure**	**Target**	**$\lambda_{abs/em}$ (nm)**	**Ref**
BODIPY-MMP	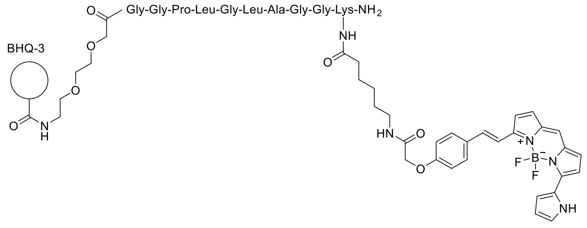	MMP-2/9	650/665	83
MMP-P12	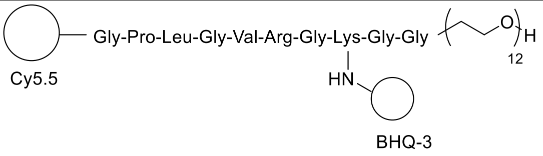	MMP2/9	683/703	84
RACPP1	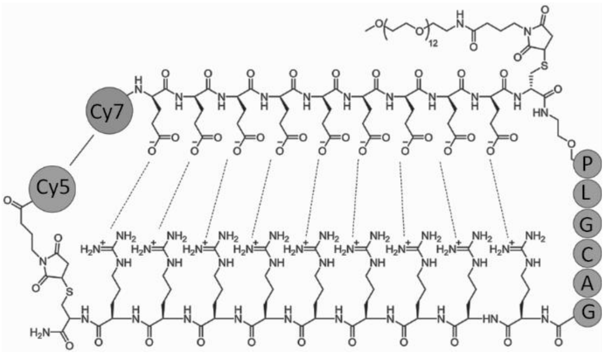	MMP-2/9 and NE	620/ 670	87
cRGD-QC	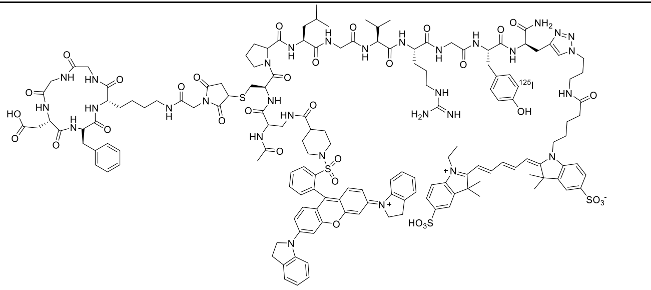	MMP-2	646/ 660	88
QC	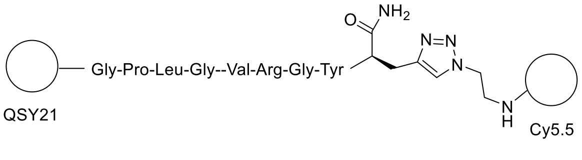	MMP-2	680/ 705	89

N/A	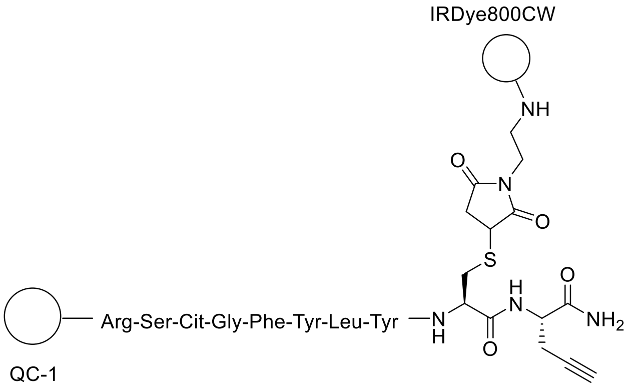	MMP-14	778/794	90
TER-SA	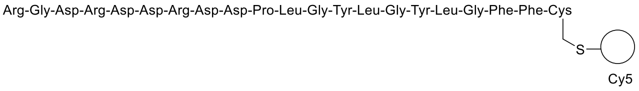	MMP-2/9	678/694	91

Table S4. Code, chemical structure, target enzyme, absorbance/emission wavelengths and reference number of probes for cysteine proteases.

Code	Structure	Target	$\lambda_{abs/em}$ (nm)	Ref
C-SNAF	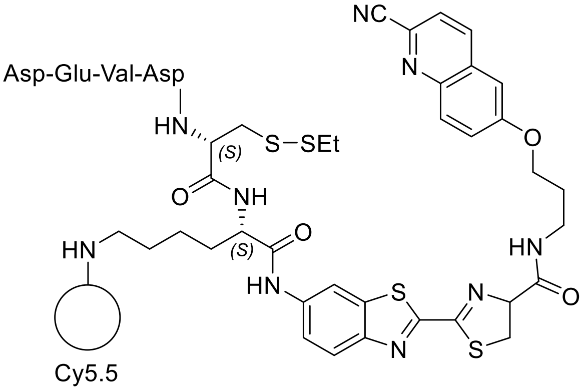	Casp-3/7	683/703	97
Mc-Probe	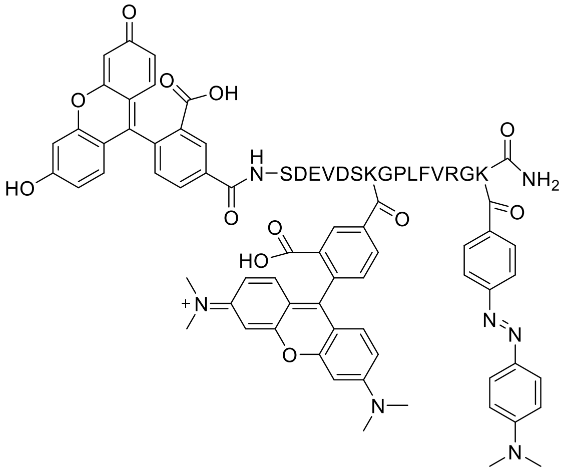	Casp-3 / MMP-2	variable	98
CFR	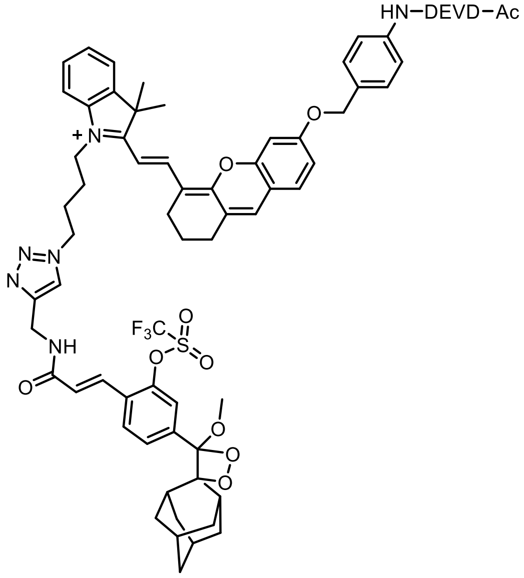	Casp-3	680/710	99

MRP3	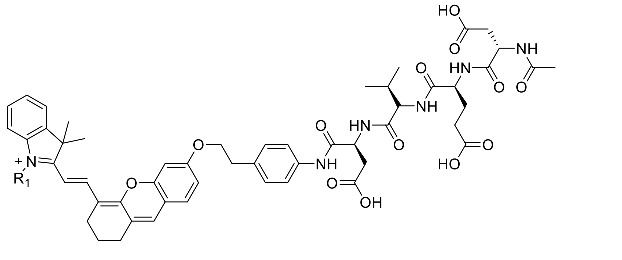	Casp-3	680/710	100
Ac-Tat-DEVD-CV	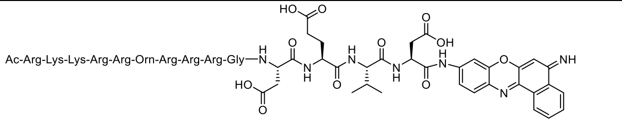	Casp-3	586/628	101
Cas-1	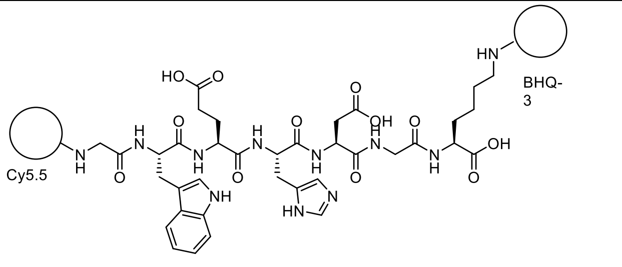	Casp-1	683/703	102
BMV083	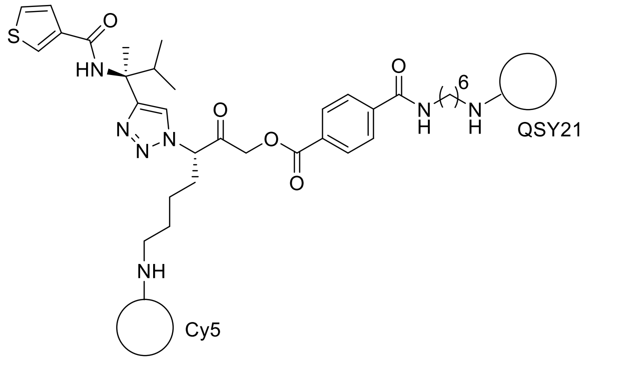	Cath S	649/666	107
BMV109	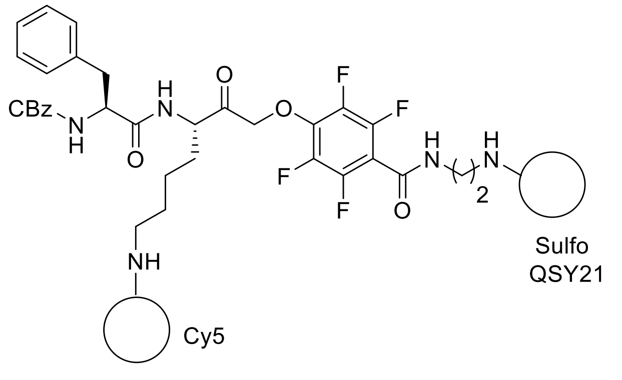	Pan-Cath	649/666	108
sCy5-Nle-SY	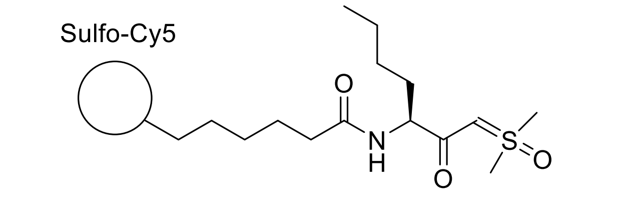	Cath X	649/666	110

BMV157	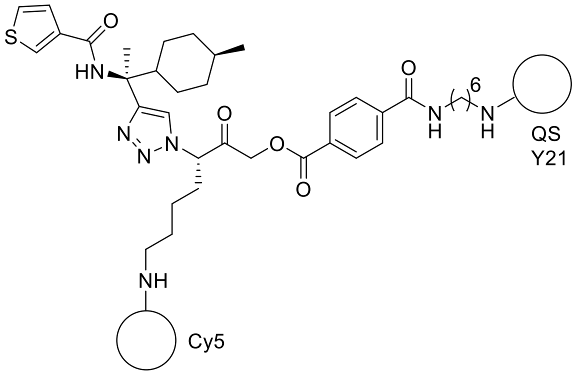	Cath S	649/666	111
VGT309	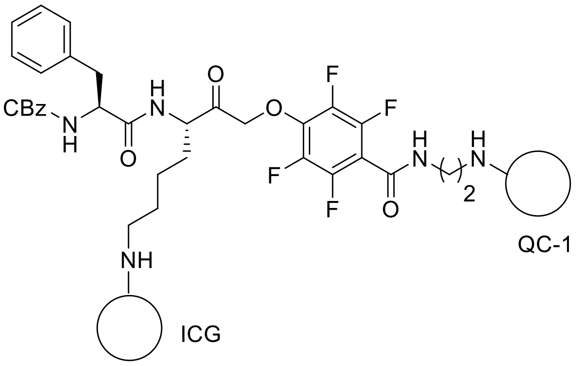	Cath S	789/814	112
MP-cL3	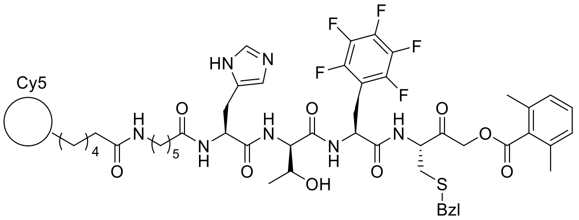	Cath L	649/666	113
MP-cB-2	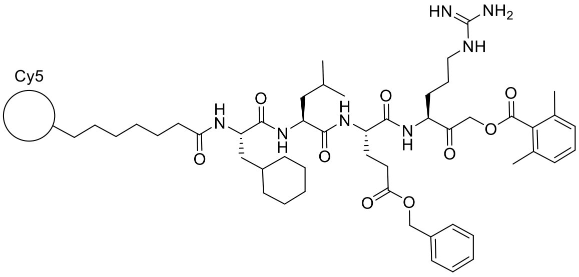	Cath B	649/666	114

LUM015	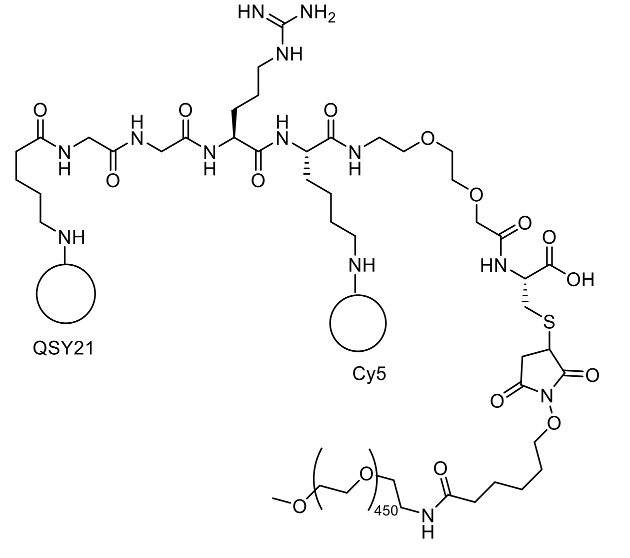	Pan-Cath	649/666	116
6QCNIR	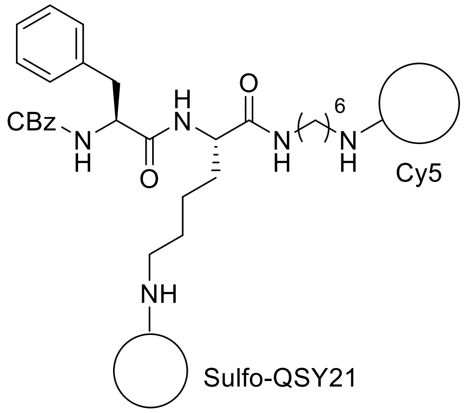	Pan-Cath	649/666	117
CyA-P-CyB	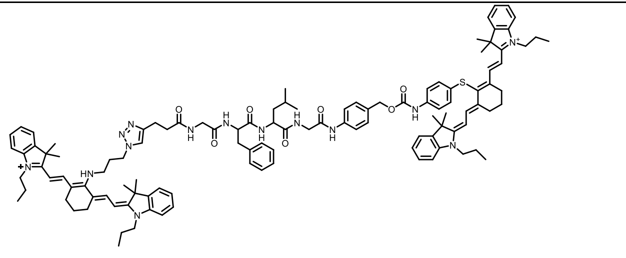	Cath B	690/720	120
